# Insular responses to transient painful and non-painful thermal and mechanical spinothalamic stimuli recorded using intracerebral EEG

**DOI:** 10.1038/s41598-020-79371-2

**Published:** 2020-12-18

**Authors:** Giulia Liberati, Dounia Mulders, Maxime Algoet, Emanuel N. van den Broeke, Susana Ferrao Santos, José Géraldo Ribeiro Vaz, Christian Raftopoulos, André Mouraux

**Affiliations:** 1 Institute of Neuroscience, Université catholique de Louvain, Brussels, Belgium; 2 Department of Neurology, Saint-Luc University Hospital, Brussels, Belgium; 3 Department of Neurosurgery, Saint-Luc University Hospital, Brussels, Belgium

**Keywords:** Somatosensory system, Neuroscience, Sensory processing

## Abstract

Brief thermo-nociceptive stimuli elicit low-frequency phase-locked local field potentials (LFPs) and high-frequency gamma-band oscillations (GBOs) in the human insula. Although neither of these responses constitute a direct correlate of pain perception, previous findings suggest that insular GBOs may be strongly related to the activation of the spinothalamic system and/or to the processing of thermal information. To disentangle these different features of the stimulation, we compared the insular responses to brief painful thermonociceptive stimuli, non-painful cool stimuli, mechano-nociceptive stimuli, and innocuous vibrotactile stimuli, recorded using intracerebral electroencephalograpic activity in 7 epileptic patients (9 depth electrodes, 58 insular contacts). All four types of stimuli elicited consistent low-frequency phase-locked LFPs throughout the insula, possibly reflecting supramodal activity. The latencies of thermo-nociceptive and cool low-frequency phase-locked LFPs were shorter in the posterior insula compared to the anterior insula, suggesting a similar processing of thermal input initiating in the posterior insula, regardless of whether the input produces pain and regardless of thermal modality. In contrast, only thermo-nociceptive stimuli elicited an enhancement of insular GBOs, suggesting that these activities are not simply related to the activation of the spinothalamic system or to the conveyance of thermal information.

## Introduction

Studies using intracerebral electroencephalography (iEEG) have shown that brief thermo-nociceptive stimuli elicit at least two functionally distinct types of responses in the human insula, namely low-frequency phase-locked local field potentials (LFPs)^1–3^ and gamma-band oscillations (GBOs, 40–90 Hz)^4^. However, neither of these activities can be considered as a direct correlate of pain perception. Low-frequency phase-locked LFPs recorded from the insula can also be elicited by non-painful vibrotactile, auditory, and visual stimuli provided that they are intense, and therefore probably reflect supramodal activity that is not specific for nociception and pain^3–5^. Insular GBOs do appear to be preferentially elicited by nociceptive heat stimuli^4^, but their response magnitude can be dissociated from the intensity of pain perception, e.g. when the nociceptive stimuli are delivered at a constant and predictable time interval^5^.

In primates, tracing studies have shown that the posterior insula and the medial parietal operculum are important targets for inputs ascending the spinothalamic tract, known to be a major route for transmitting information about both noxious and innocuous thermoception^6^. In humans, this notion is supported by evidence from functional neuroimaging studies^7–10^, lesion studies^11^, intracerebral recordings^12^, and direct stimulation^13^. In this study, we aimed to investigate whether transient insular responses elicited by brief thermo-nociceptive stimuli—and particularly insular GBOs—were related to the activation of specific types of inputs conveyed by the spinothalamic system.

**Figure 1:**
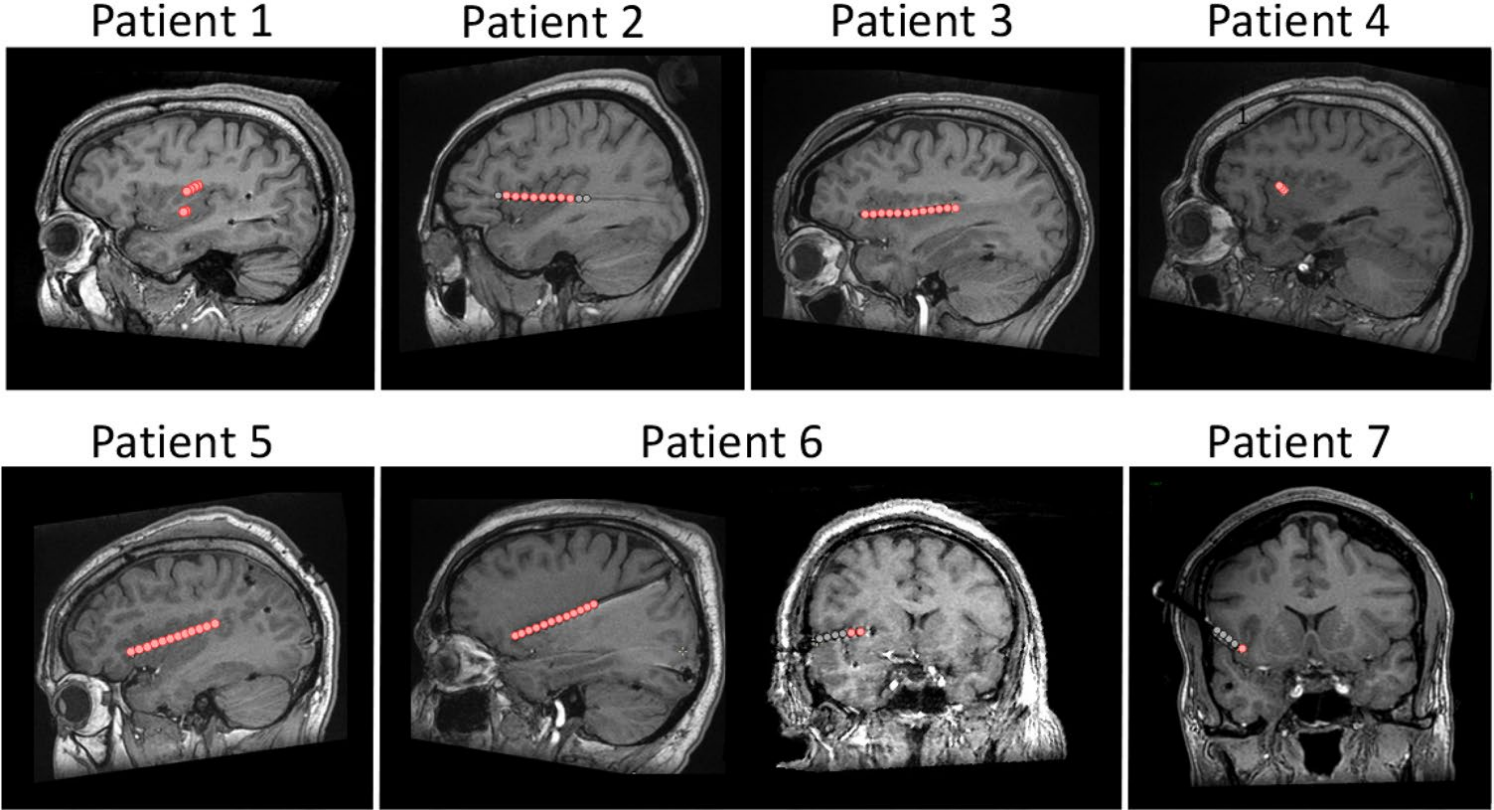
Localization of 9 depth electrodes implanted in the insula of 7 patients. A total of 58 insular sites (32 in the anterior insula, 26 in the posterior insula; 31 in the left insula, 27 in the right insula; indicated by red circles) were investigated. The locations and MNI coordinates of the electrode contacts are presented in Table 2 and in Suppl 1. The DICOM images of each patient are available at the OSF online repository at the address https://osf.io/tzqru/.

**Figure 2:**
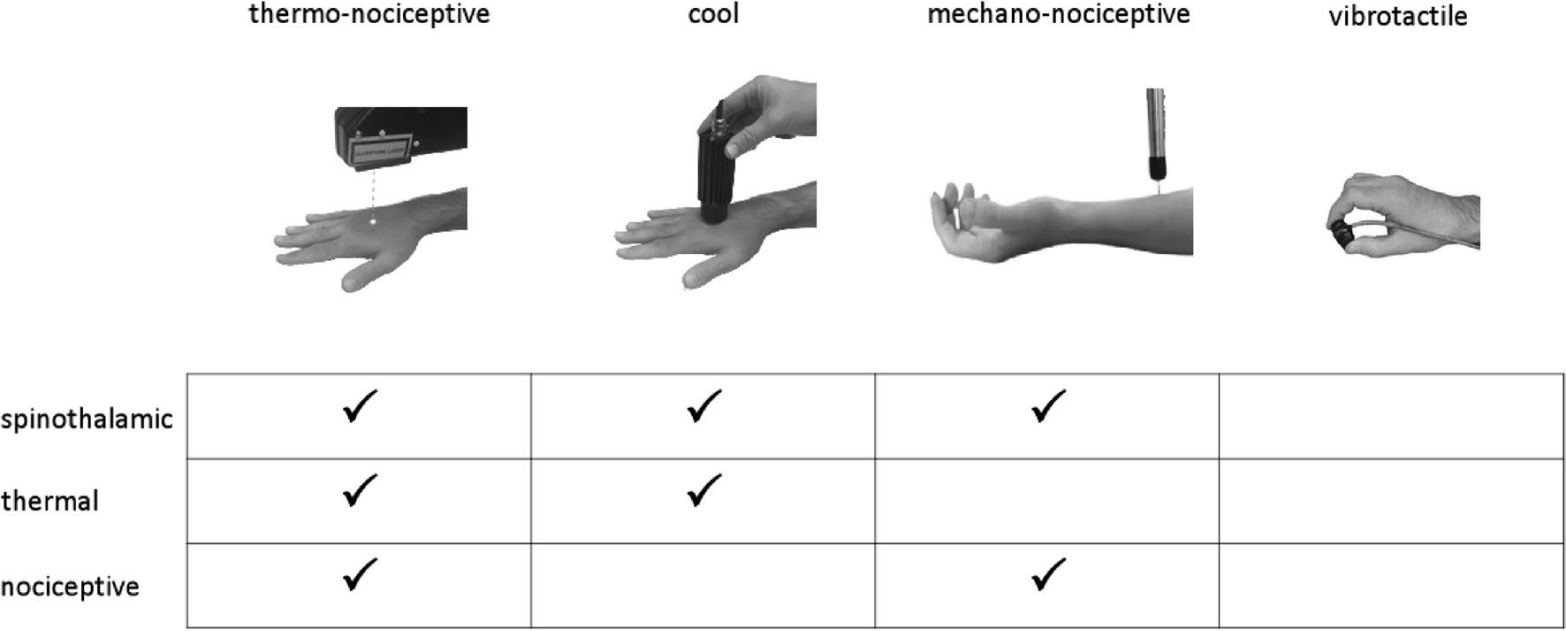
Sensory stimulation. Participants received four types of stimuli, in a block design. Painful thermo-nociceptive stimuli consisted of brief pulses of radiant heat applied to the hand dorsum using a temperature-controlled CO_2_ laser, activating heat-sensitive nociceptors. Cool stimuli were delivered using a thermal cutaneous stimulator device activating cool-sensitive free nerve endings. Pinprick stimuli activating mechano-sensitive nociceptors were delivered using a custom-built pinprick device, constituted by a holding cylinder containing a freely-sliding steel flat tip probe with a calibrated cylindrical weight on top. Vibrotactile stimuli, activating mechanoreceptors of the medial lemniscus system, were short-lasting mechanical vibrations delivered to the thumb and index fingertips.

Using iEEG recorded from 7 patients undergoing a presurgical evaluation of focal epilepsy (Fig. 1), we aimed to differentiate activities elicited in the human insula by somatosensory stimuli varying along different dimensions, such as the activation of nociceptors, the activation of the spinothalamic system, and the conveyance of thermal (heat or cold) information. To this end, we compared insular responses to brief stimuli belonging to the following four modalities (Fig. 2): (i) painful thermo-nociceptive stimuli activating heat-sensitive nociceptors; (ii) non-painful cool stimuli activating cool-sensitive free nerve endings; (iii) non-painful pinprick stimuli activating mechano-sensitive nociceptors; and (iv) innocuous vibrotactile stimuli activating low-threshold mechanoreceptors of the medial lemniscus system. To ensure that the stimuli belonging to the four different modalities did not differ markedly in terms of perceived intensity, participants were asked to provide intensity ratings on a numerical rating scale. In addition, to ensure that the salience of the different stimuli (i.e., the ability of a stimulus to stand out in the sensory environment^14,15^) was comparable across modalities, we computed stimulus-evoked heart rate change, associated with activation of the autonomic system, as a surrogate measure of stimulus salience^16^. Finally, because of the evidence that distinct subregions of the insula may respond differentially to nociceptive and non-nociceptive stimuli^17–21^, and to assess possible lateralization effects^22–25^, we compared responses recorded from the anterior and posterior portions of the insula, as well as responses recorded from the left and right insula.

## Results

### Quality and intensity of perception

All participants described thermo-nociceptive stimuli, but not the other stimuli, as clearly painful and pricking. Mechano-nociceptive stimuli were described as slightly pricking, and both cool and vibrotactile stimuli were described as intense but non-painful. The ratings of intensity did not differ significantly across modalities (Kruskall–Wallis H test: χ^2^ = 1.51, p = 0.681), although mechano-nociceptive stimuli (3.1 ± 1.2) were on average rated as slightly less intense than thermo-nociceptive (4.5 ± 2.4), cool (4.4 ± 2.6), and vibrotactile (4.8 ± 2.2) stimuli.

### Heart rate change

Regardless of stimulation modality, salient stimuli are known to trigger autonomic responses^16^. Hence, to ensure that none of the stimuli were perceived as markedly more salient than the others, we computed stimulus-evoked heart rate change as an indirect measure of stimulus-evoked salience. A linear mixed models (LMM) analysis of the heart rate change using ‘modality’ (4 levels: thermo-nociceptive, cool, mechano-nociceptive, vibrotactile) and ‘time interval’ (2 levels: 5 s preceding stimulation, 5 s following stimulation) as factors showed a significant effect of ‘time interval’ (F = 6.3, p = 0.016). On average, sensory stimulation triggered an increase of heart rate change in all four modalities (thermo-nociceptive: 0.9 ± 1.0 bpm; cool: 0.8 ± 1.0 bpm; mechano-nociceptive: 1.9 ± 2.6 bpm; vibrotactile: 1.3 ± 2.8). In contrast, there was no effect of ‘modality’ (F = 0.7, p = 0.574), and no interaction between ‘modality’ and ‘time interval (F = 0.3, p = 0.858).

### Intracerebral responses recorded from the insula

**Figure 3:**
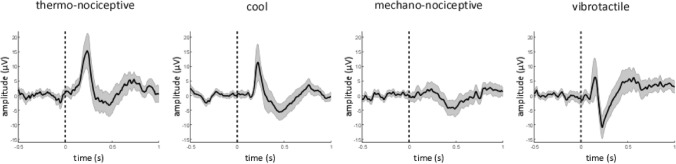
Low-frequency phase-locked local field potentials (LFPs) recorded from the insula. All four types of stimuli elicited low-frequency phase-locked LFPs in the insula, appearing as biphasic waves (group level average; confidence intervals stated at the 95% confidence level are shown in gray). The amplitudes of thermo-nociceptive, cool, and vibrotactile low-frequency phase-locked LFPs were significantly greater than the amplitudes of mechano-nociceptive low-frequency phase-locked LFPs.

**Figure 4:**
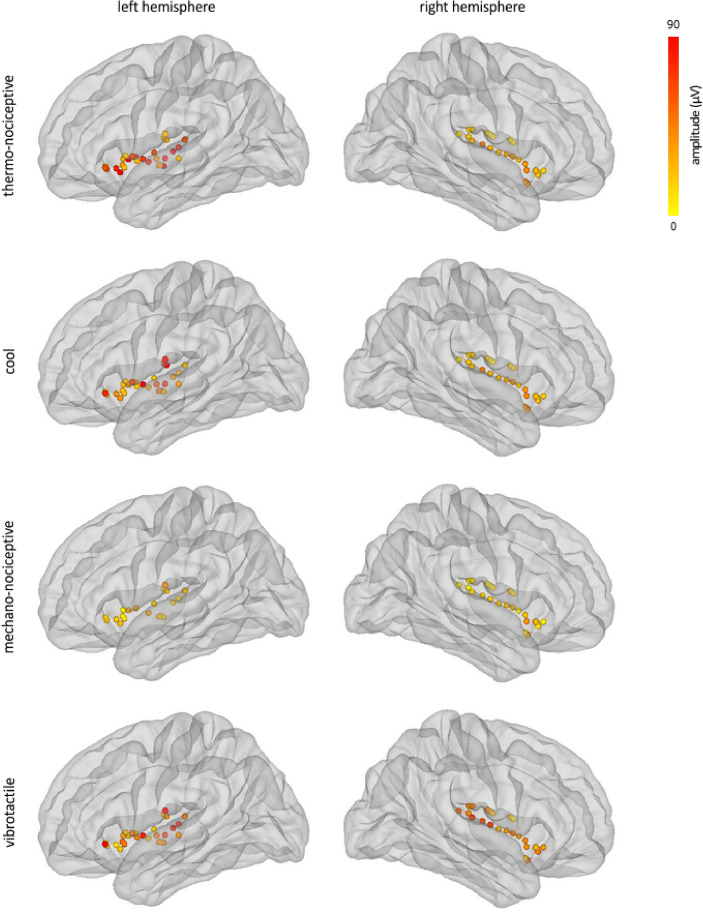
Spatial distribution of the amplitudes of low-frequency phase-locked LFPs elicited by thermo-nociceptive, cool, mechano-nociceptive, and vibrotactile stimuli, in the left and right insula. The circles correspond to the insular contacts of all patients, within 3D glass brains generated using the FreeSurfer template. For the thermo-nociceptive and cool modalities, the amplitudes of low-frequency phase-locked LFPs were significantly greater in the left insula compared to the right insula.

All types of stimuli elicited robust low-frequency phase-locked LFPs in the insula (Fig. 3). A linear mixed model (LMM) analysis of the peak-to-peak amplitudes of low-frequency phase-locked LFPs yielded a significant effect of ‘modality’ (F = 12.8, p < 0.001), as the amplitudes of thermo-nociceptive (43 ± 30 μV), cool (38 ± 27 μV), and vibrotactile (44 ± 21 μV) low-frequency phase-locked LFPs were all significantly greater than the amplitudes of mechano-nociceptive (20 ± 9 μV) low-frequency phase-locked LFPs (p < 0.001, p = 0.001, and p < 0.001, respectively); a significant interaction between ‘modality’ and ‘side’ (F = 5.4, p < 0.001); a significant interaction between ‘modality’ and ‘location’ (F = 5.0, p = 0.002); and a significant interaction between ‘side’ and ‘location’ (F = 4.0, p = 0.047). Post-hoc comparisons showed that the amplitudes of both thermo-nociceptive and cool low-frequency phase-locked LFPs elicited by stimulation of the hand dorsum contralateral to the recording electrode were significantly greater in the left insula compared to the right insula (p = 0.002 and p = 0.008, respectively); that the amplitudes of vibrotactile low-frequency phase-locked LFPs were significantly greater in the posterior insula compared to the anterior insula (p = 0.001); and that the amplitudes of low-frequency phase-locked LFPs recorded from posterior insula were significantly greater in the left hemisphere compared to the right hemisphere (p = 0.018). The spatial distribution of the amplitudes of low-frequency phase-locked LFPs elicited by the four different modalities in the left and right insula is shown in Fig. 4.

The LMM analyses performed on the latencies of the first and second peaks of the low-frequency phase-locked LFPs showed a main effect of ‘modality’ for both peaks (peak 1: F = 104.6, p < 0.001; peak 2: F = 72.9, p < 0.001), with vibrotactile responses having the shortest latencies and cool responses having the longest latencies (see Table 1). For the first peak, the analysis also showed significant interactions between ‘modality’ and ‘side’ (F = 6.2, p < 0.001), between ‘modality’ and ‘location’ (F = 4.2, p < 0.007), and between ‘side’ and ‘location’ (F = 11.7, p = 0.001). Post-hoc comparisons showed that the latencies of the first peak of mechano-nociceptive low-frequency phase-locked LFPs were significantly shorter in the left insula compared to the right insula (p = 0.020); that the latencies of the first peaks of thermo-nociceptive and cool low-frequency phase-locked LFPs were significantly shorter in the posterior insula compared to the anterior insula (p = 0.001 for both modalities); and that in the right insula, the latencies of the first peaks were significantly shorter for posterior contacts than for anterior contacts (p < 0.001). For the second peak, the analysis showed a significant three-way interaction between ‘modality’, ‘location’, and ‘side’ (F = 3.0, p = 0.031). Post-hoc analyses showed that the latencies of the second peak of mechano-nociceptive low-frequency phase-locked LFPs recorded from the anterior insula were significantly shorter in the left insula compared to the right insula (p < 0.001).

**Table 1:** Latencies of low-frequency phase-locked local field potentials.

Modality	Peak 1 (ms)	Peak 2 (ms)
Thermo-nociceptive	224 ± 56	375 ± 85
Cool	263 ± 43	410 ± 95
Mechano-nociceptive	149 ± 73	306 ± 111
Vibrotactile	128 ± 32	222 ± 58

To verify whether the LFPs elicited by the different types of stimuli reflected modality-preferential or supramodal activities, we used a blind source separation algorithm based on independent component analysis (ICA) to break down the waveforms into a set of independent components (ICs)^26^. Each IC was classified based on its contribution to the LFPs from the different modalities, either as preferential for a specific modality (thermo-nociceptive-, cool-, mechano-nociceptive-, or vibrotactile-preferential), preferential for thermal input (thermo-nociceptive and cool), preferential for spinothalamic input (thermo-nociceptive, cool, and mechano-nociceptive), preferential for nociceptive input (thermo-nociceptive and mechano-nociceptive stimuli), preferential for mechanical input (mechano-nociceptive and vibrotactile) or supramodal. Patient 7, who had only one electrode contact located in the insula, was excluded from the ICA analysis. The estimated number of independent sources contributing to the four LFP waveforms (thermo-nociceptive, cool, mechano-nociceptive, and vibrotactile) ranged, across insulae, between 6 and 12 (9 ± 4). The large majority of ICs were classified as supramodal (75%), i.e. contributing to the responses elicited by all four types of stimuli. The remaining ICs were classified either as vibrotactile (10%), thermo-nociceptive (4%), cool (4%), mechanoceptive (i.e. mechano-nociceptive + vibrotactile, 4%), or thermal (i.e. thermo-nociceptive + cool, 2%). None of the components were classified as nociceptive (thermo-nociceptive + mechano-nociceptive) or as mechano-nociceptive. Supramodal ICs were the main constituent of all LFPs, explaining, across patients, 92 and 70% of the thermo-nociceptive peaks, 78 and 76% of the cool peaks, 55 and 58% of the mechano-nociceptive peaks, and 57 and 68% of the vibrotactile peaks, compatible with the notion that low-frequency phase-locked LFPs recorded from the human insula largely reflect the same source of supramodal cortical activity^3^.

**Figure 5:**
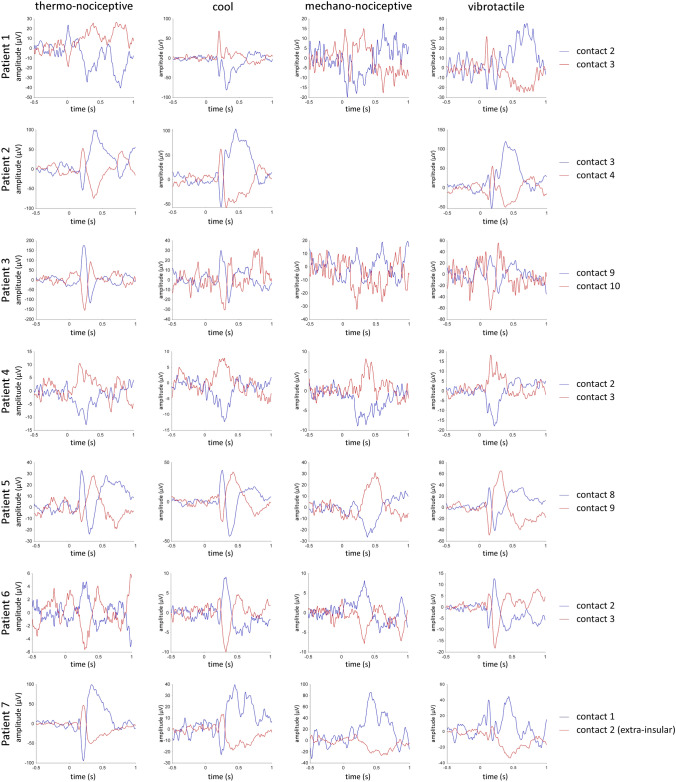
Phase reversals in low-frequency phase-locked LFPs recorded from the insula. For each patient, examples of phase reversal in the responses to thermo-nociceptive, cool, mechano-nociceptive and vibrotactile stimulation are presented. The signal measured at each insular contact is displayed using the average of the two adjacent contacts as a reference (linear current source density). Because the electrical activity generated in a given area can be summarized as an equivalent current dipole located close to the center of activity with an orientation orthogonal to the activated cortical surface, contacts showing a phase reversal may be considered as located closest to a source of activity, respectively in front and behind the dipole source. The locations of the reversals were often overlapping across the different modalities, suggesting a common source for the generated activity.

To examine whether the observed low-frequency phase-locked LFPs originated within the insular cortex instead of being volume-conducted from extra-insular regions, we identified phase reversals in the insulae of all participants. To better visualize the phase reversals, the signal measured at each insular contact was displayed using the average of the two adjacent contacts as a reference (linear current source density)^3^ (Fig. 5). The locations of the reversals were largely shared across the different modalities of stimulation, suggesting a common source for the generated activity.

**Figure 6:**
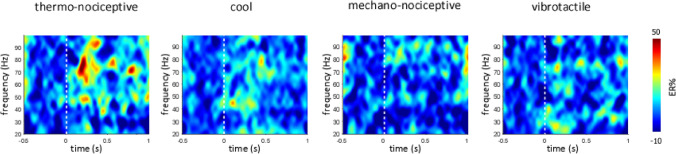
Time–frequency representation of the changes in oscillatory power (20–100 Hz) recorded at the insular contacts in which, for each patient, GBOs elicited by thermo-nociceptive stimuli were more pronounced (group level percentage of change, ER%). As compared to cool, mechano-nociceptive and vibrotactile stimuli, thermo-nociceptive stimuli elicited a greater increase in GBO power. Single-subject GBO responses elicited by thermo-nociceptive stimuli at the indicated insular locations are shown in Suppl. 2.

**Figure 7:**
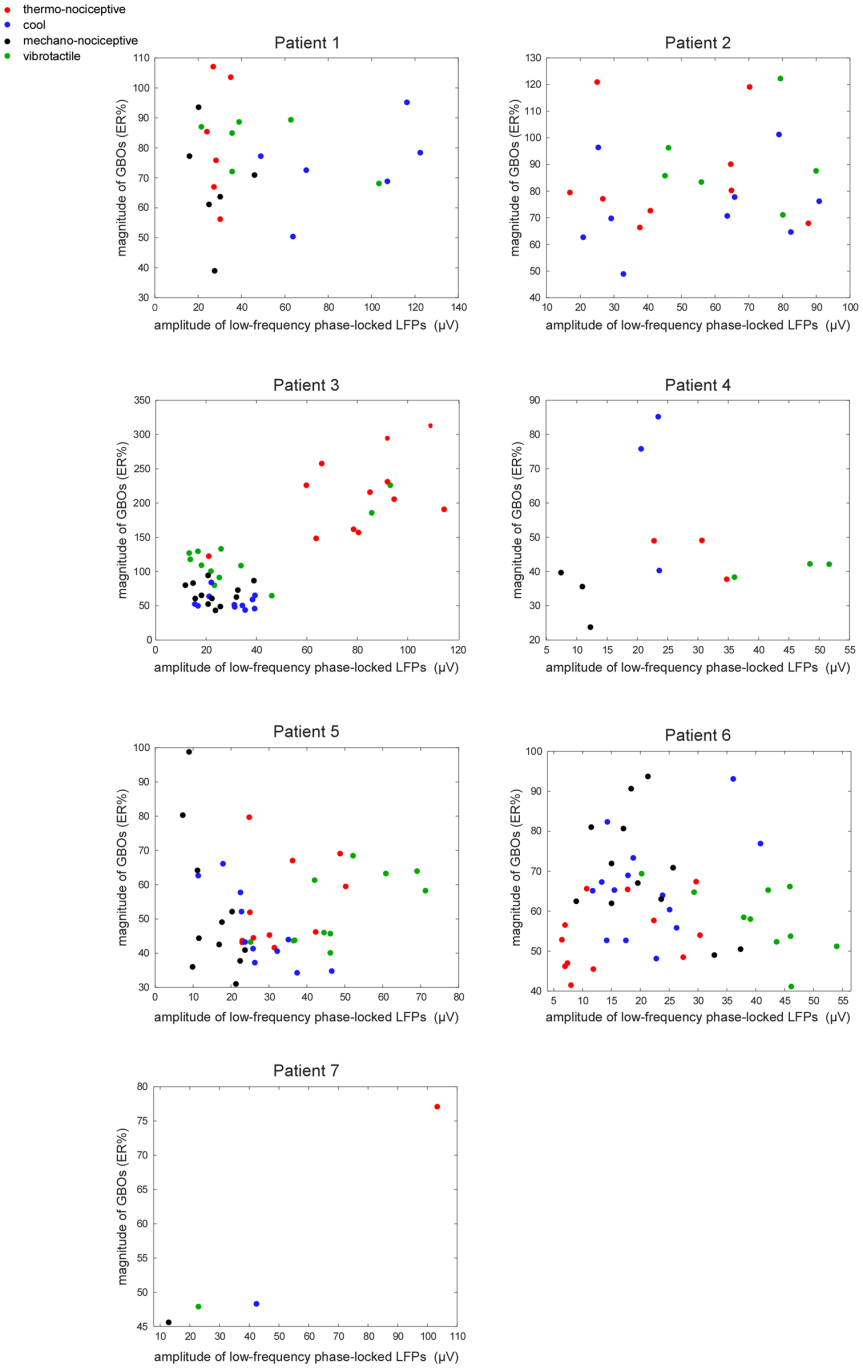
Dissociation between low-frequency phase-locked LFPs and GBOs recorded from the human insula. For each participant, the amplitudes of the responses to thermo-nociceptive, cool, mechano-nociceptive, and vibrotactile stimuli, recorded at each insular contact, are displayed. X-axes: amplitudes of low-frequency phase-locked LFPs. Y-axes: maximum post-stimulus change in GBO power and expressed as percentage of change relative to baseline (ER%), considering the 40–90 Hz frequency range and the 150–300 ms post-stimulus time interval. The dissociation between GBOs (exhibiting a strong increase only following nociceptive stimulation) and low-frequency phase-locked LFPs (presenting similar magnitudes across modalities) suggests that these two insular activities reflect different neural processes.

**Figure 8:**
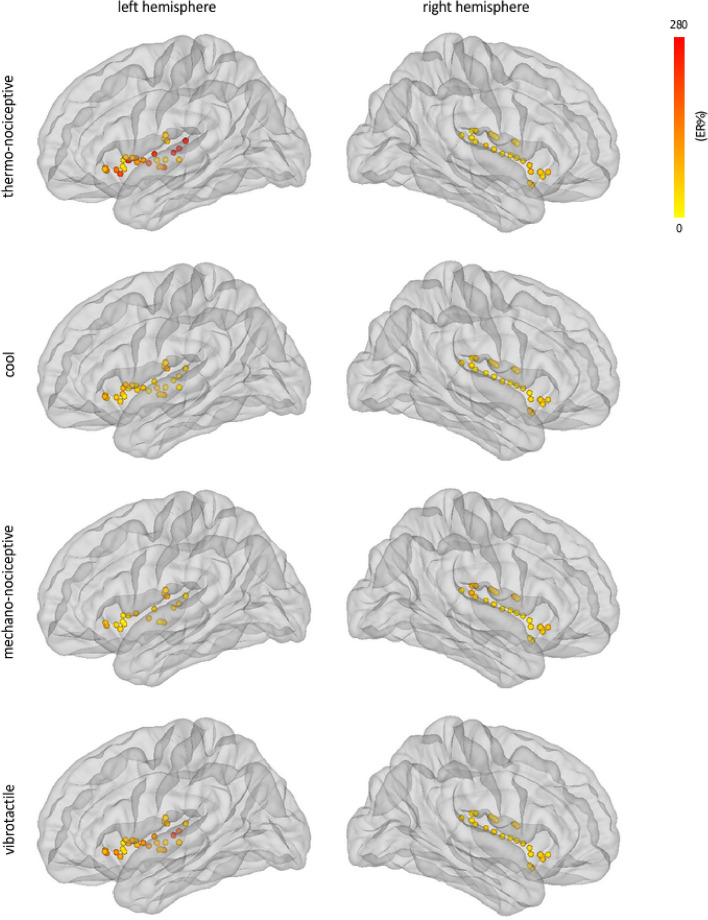
Spatial distribution of the magnitudes GBOs elicited by thermo-nociceptive, cool, mechano-nociceptive, and vibrotactile stimuli, in the left and right insula, considering the 40–90 Hz frequency range and the 150–300 ms post-stimulus time interval. The circles correspond to the insular contacts of all patients, within 3D glass brains generated using the FreeSurfer template (http://surfer.nmr.mgh.harvard.edu/). In the left insula, but not in the right insula, the magnitudes of thermo-nociceptive GBOs were significantly greater than the magnitudes of cool, mechano-nociceptive, and vibrotactile GBOs.

Thermo-nociceptive stimuli elicited an early-latency (150–300 ms) enhancement of GBOs (40–90 Hz), which was not observed in response to cool, mechano-nociceptive, and vibrotactile stimuli (Fig. 6). The LMM analysis performed on the GBO amplitudes yielded a main effect of ‘location’ (F = 4.8, p = 0.030), as GBOs recorded from the posterior insula were on average greater in amplitude than GBOs recorded from the anterior insula; and a significant interaction between ‘side’ and ‘modality’ (F = 17.4, p < 0.001). Post-hoc comparisons showed that in the left insula, but not in the right insula, the amplitudes of thermo-nociceptive GBOs (133 ± 78 ER%) were significantly greater in amplitude than the amplitudes of cool (66 ± 17 ER%), mechano-nociceptive (63 ± 20 ER%), and vibrotactile (96 ± 40 ER%) GBOs (all p < 0.001). The dissociation between the elicited GBOs and the low-frequency phase-locked LFPs recorded at the same insular contacts is shown in Fig. 7. The spatial distribution of the amplitudes of GBOs elicited by the four different modalities in the left and right insula is shown in Fig. 8.

## Discussion

Brief thermo-nociceptive, cool, mechano-nociceptive, and vibrotactile stimuli all elicited low-frequency phase-locked LFPs, appearing as large biphasic waves, widespread throughout the anterior and posterior insula. Although the spatial resolution and sampling of LFPs do not allow differentiating responses generated by individual neurons or small anatomical loci, and although low-frequency phase-locked LFPs may comprise modality-specific contributions that are undetected at macroscopic level, these findings are consistent with our previous observations that different types of transient stimuli elicit low-frequency phase-locked LFPs in the human insula—of the same order of magnitude across modalities—regardless of painfulness and nociception^3–5^. The identification of phase reversals in all of the participants indicates that these responses were largely originating from within the insula. Interestingly, the locations of the phase reversals were mostly shared across modalities of stimulation, suggesting a common source for insular low-frequency phase-locked LFPs—an observation that is consistent with a previous study from our group. Furthermore, a blind source separation algorithm confirmed that these low-frequency phase-locked LFPs can be largely explained by a common supramodal source of insular activity.

The across-modality differences in the latency of low-frequency phase-locked LFPs can be explained by the difference in time required for the sensory afferent volleys to reach the cortex^27,28^. In particular, the greater latency of thermo-nociceptive and cool low-frequency phase-locked LFPs as compared to vibrotactile low-frequency phase-locked LFPs are explained by the fact that small-diameter A-delta fibers conveying spinothalamic input have a slower conduction velocity than large-diameter A-beta fibers conveying vibrotactile input. On average, low-frequency phase-locked LFPs elicited by mechano-nociceptive stimuli showed shorter latencies compared to thermo-nociceptive and cool low-frequency phase-locked LFPs, but longer latencies compared to vibrotactile low-frequency phase-locked LFPs—a difference that may be explained by the fact that mechano-nociceptive stimuli activated both A-beta and A-delta fibers. An alternative explanation is suggested by a recent microneurography investigation performed on cutaneous mechanoreceptor afferents in healthy participants, which identified a new class of A-fiber high-threshold mechanoreceptors, insensitive to gentle touch and encoding noxious skin indentations, which displayed conduction velocities similar to A-fiber low-threshold mechanoreceptors^29^.

Thermal low-frequency phase-locked LFPs (thermo-nociceptive and cool), but not mechanical low-frequency phase-locked LFPs (mechano-nociceptive and vibrotactile), were on average shorter in latency in the posterior insula compared to the anterior insula. Although we previously did not observe a difference in latency between anterior and posterior thermo-nociceptive responses^3^—most likely due to the limited number of contacts located in the posterior insula, this difference was reported by Frot et al.^30^, who concluded that painful nociceptive stimuli are first processed in the posterior insula and then conveyed to the anterior insula. Our observation suggests that this interpretation may be extended to thermal stimuli in general, regardless of their being nociceptive and/or painful.

In addition to low-frequency phase-locked LFPs, brief thermo-nociceptive stimuli elicited an early latency (150–300 ms) enhancement of GBOs (40–90 Hz) in the left insula. In contrast, non-painful cool, mechano-nociceptive, and vibrotactile stimuli did not elicit a comparable increase of GBOs, corroborating our previous observations that GBOs recorded from the human insula are both selective for thermonociception and dissociated from low-frequency phase-locked LFPs^4,5^. It could be argued that the preferential enhancement of GBOs following thermo-nociceptive stimuli compared to cool, mechano-nociceptive, and vibrotactile stimuli may be due to a gross difference in the intensity and/or saliency of the stimulation. On average, the intensity ratings provided by participants were not significantly different across modalities, suggesting that none of the four types of stimuli was systematically perceived as more intense than the others. Moreover, the four types of stimuli led to a similar heart rate change, associated with autonomic nervous system arousal and used as an indirect measure of salience^16^, given that stimuli that exhibit characteristics that could make them perceived as more salient (e.g., high intensity, high distinction from the surroundings) also elicit greater autonomic responses. Notably, heart rate changes are likely to reflect both sympathetic and parasympathetic activities of the autonomic nervous system, which can operate reciprocally, in parallel, or independently. Hence, a more precise measure of the construct of arousal should be gained, in future studies, by combining different autonomic measures across different end-organs (e.g. heart rate, skin conductance, pupil diameter)^16^.

The strongest evidence that the preferential enhancement of GBOs following thermo-nociceptive stimuli was not due to a gross difference in the intensity of the different stimuli is that all four types of stimuli elicited consistent low-frequency phase-locked LFPs, which were of the same order of magnitude for thermo-nociceptive, cool, and vibrotactile stimuli. Mechano-nociceptive stimuli, which were also rated as slightly less intense than the other types of stimuli, elicited smaller low-frequency phase-locked LFPs—an indication that these stimuli might have been less intense and/or salient compared to the other stimuli.

Because both cool stimuli and mechano-nociceptive stimuli activate the spinothalamic system, it appears that spinothalamic activation per se is not sufficient to elicit a marked increase of GBOs in the human insula. Indeed, the mechano-nociceptive stimuli used in this study might have not been sufficiently intense to elicit an increase of insular GBOs, as suggested by the lower intensity ratings provided by the participants, as well as by the fact that low-frequency phase-locked LFPs elicited by mechano-nociceptive stimuli were smaller in amplitude compared to the low-frequency phase-locked LFPs elicited by the stimuli in the other modalities, possibly also due to a lower spatial summation of the stimulation, considering that the probe surface was rather small. However, this argument does not apply to the cool stimuli, which elicited responses that were comparable to thermo-nociceptive stimuli in terms of both intensity ratings and magnitude of the elicited low-frequency phase-locked LFPs. Hence, the mere conveyance of thermal information does not appear to be sufficient per se to elicit a marked increase of GBOs in the human insular cortex.

Although we had already recorded iEEG responses to brief thermo-nociceptive stimuli from both the left and the right insula in two previous studies^3,4^, this is the first study in which we were able to record activities from a sufficient number of electrode contacts located in the right insula, which allowed us to assess hemispheric differences. Interestingly, the amplitudes of low-frequency phase-locked LFPs elicited by both types of thermal stimuli (thermo-nociceptive and cool) were significantly greater in the left insula compared to the right insula. Moreover, GBOs elicited by thermo-nociceptive stimuli were also more prominent in the left insula compared to the right insula. These observations suggest a possible left hemisphere bias in the processing of thermal stimuli. A similar left hemisphere bias in the activation of the operculoinsular cortex in response to thermo-nociceptive stimulation was observed in scalp EEG and source localization studies^22^, as well as in functional magnetic resonance imaging (fMRI)^23^ studies. However, in contrast with our findings, another fMRI study^24^ showed a right-sided lateralization of the insula in the processing of thermo-nociceptive stimuli, whereas no insular lateralization of thermo-nociceptive processing emerged from a positron emission tomography (PET) study^25^. It is also worth noting that due to the arrangement of the hospital room where presurgical evaluations of epileptic patients were performed in the current study, all the equipment for stimulation was systematically located on the right side of the patient—a setup that might have contributed to the current findings, albeit this effect was never systematically studied. To this end, further investigations of the differential roles of the right and left insula in somatosensation, nociception and pain perception should be conducted, also including larger sample sizes.

Overall, whereas all four types of stimuli elicited consistent low-frequency phase-locked LFPs throughout the insula, thermal (thermo-nociceptive and cool) low-frequency phase-locked LFPs shared a number of characteristics, including shorter latencies in the posterior insula compared to the anterior insula, and a possible lateralization (i.e. greater amplitudes in the left insula). These similarities suggest a common processing of thermal stimuli—regardless of painfulness and nociception—initiating in the posterior insula. These findings are also consistent with the proposition that the posterior insula, and particularly its most dorsal portion, comprises a “primary interoceptive cortex”, which processes information concerning the physiological condition of all the organs and tissues of the body, including skin temperature^18,31,32^. Differently from cool stimuli, however, thermo-nociceptive stimuli elicited an enhancement of insular GBOs, supporting the conclusion that these activities are not simply related to the activation of the spinothalamic system or to the conveyance of thermal information, but may be preferential for nociception, despite not constituting a direct measure of pain. Further investigations comparing insular responses to brief thermo-nociceptive stimuli with insular responses to brief mechano-nociceptive stimuli of higher intensity than the ones used in the current study (i.e. eliciting low-frequency phase-locked LFPs of comparable amplitude) could allow understanding whether insular GBOs are preferential for nociception in general, or whether they also depend on the thermal quality of the nociceptive input.

## Methods

### Participants

We recruited 7 patients (2 females; age: 24–53) undergoing a presurgical evaluation of intractable epilepsy at the Department of Neurology of Saint Luc University Hospital (Brussels, Belgium). Patients 1–5 also participated in other experiments conducted by our team^4,5,33^. As in previous studies from our group^3–5,33^, all patients were investigated using depth electrodes implanted in various brain regions suspected to be the origins of seizures, including wide portions of the anterior and posterior left and right insulae (Fig. 1 and Table 2; see Suppl. 1 for the MNI coordinates of each electrode contact). Intracerebral EEG was recorded from 9 insular depth electrodes (Patients 1 and 6 were each implanted with two distinct insular electrodes), for a total of 58 contacts (31 left, 27 right; 32 in the anterior insula and 26 in the posterior insula). The anterior insula was identified as the region encompassing the short insular gyri (anterior, middle, and posterior), the pole of the insula, and the transverse insular gyrus. The posterior insula was identified as the region encompassing the anterior and posterior long insular gyri^34^. Two contacts located in the left posterior insula (Patient 4) and three contacts located in the right anterior insula (Patients 5 and 6) were excluded from the analyses due to strong signal artifacts. Patient 2 did not undergo one of the stimulation blocks (mechano-nociceptive stimulation) due to a headache related to the surgical intervention. The anonymized DICOM images of each patient are available at the OSF online repository at the address https://osf.io/tzqru/. These images do not include facial features that could lead to the identification of the participants. None of the participants had psychiatric issues, cognitive impairment, or sensory abnormalities. None of the participants presented ictal discharge onset in the insula during the recordings, and low-voltage fast activity was never present in the insula during spontaneous seizure. All experimental procedures were approved by the local Research Ethics Committee (Commission Ethique de l’Université catholique de Louvain, B403201316436). All methods were performed in accordance with the relevant guidelines and regulations. Informed consent was obtained from all participants. The manuscript does not contain information or images that could lead to the identification of the participants.

**Table 2:** Number and location of insular electrode contacts.

Subject	Hemisphere	Number of contacts	Location
1	Left	6	Posterior insular cortex/operculum
2	Left	8	Anterior and posterior insular cortex
3	Left	12	Anterior and posterior insular cortex
4	Left	3	Anterior insular cortex
5	Right	11	Anterior and posterior insular cortex
6	Right	12	Anterior and posterior insular cortex
7	Right	1	Anterior insular cortex

MNI coordinates of individual electrode contacts are reported in Suppl. 1.

### Intracerebral EEG recordings

Intracerebral EEG recordings were performed using the same procedures as in our previous studies^3–5,33^. For each patient, a tailored implantation strategy was planned on the basis of the regions considered most likely to be ictal onset sites or propagation sites. The desired targets were reached using commercially available depth electrodes (AdTech, Racine, Wisconsin, United States; rod diameter: 1.1 mm; contact length: 2.4 mm; contact spacing: 5 mm) implanted using a frameless stereotactic technique through burr holes. The placement was guided by a neuronavigation based on a 3D T1-weighted MRI sequence performed in a 1.5 T scanner (Gradient Echo: flip angle: 15°; TR: 7.5 s; TE minimum full; 3.1–13 ms; slice thickness: 1 mm; FOV: 24 cm; matrix: 224 × 224; number of slices: 162). A post-implantation 3D-T1 weighted MRI sequence, performed either right after the surgery or on the following day, was used to accurately identify single contact localizations. To obtain the MNI coordinates of each insular electrode contact, we normalized individual MRI scans to a standard T1 template in MNI space using BrainVoyager 20.2 (Brain Innovation, Maastricht, The Netherlands). To visualize the spatial distributions of the response amplitudes at each contact location in the left and right insula, we then used the MNI coordinates to generate group-level 3D glass brain plots using the iELVis open source Matlab toolbox^35^ and a FreeSurfer anatomical atlas (http://surfer.nmr.mgh.harvard.edu/), after performing nonlinear mapping between the MNI volumetric coordinate system and the FreeSurfer surface coordinate system^36^.

The intracerebral EEG recordings were performed using a Deltamed (Paris, France) acquisition system. Additional bipolar channels were used to record electromyographic (EMG) activity (two electrodes measuring respectively bicipital and tricipital contraction of the non-dominant arm) and electrocardiographic (EKG) activity (two channels, utilizing two electrodes respectively located on the right and left side of the sternum, one electrode located centrally under the sternum, and one electrode located on the right lateral side of the chest). All signals were acquired at a 512 Hz sampling rate using a reference electrode located between Cz and Pz, re-referenced to the average activity recorded from all intracerebral contacts, and analyzed offline using Letswave 6^37^. Additional statistical analyses were carried out using IBM SPSS Statistics 24 (Armonk, NY).

### Experimental design

The study was conducted at the patient bedside. Before the beginning of the experiment, patients were briefly exposed to test stimuli for familiarization. The experiment consisted of four blocks, which were randomized across participants. In each block, patients received 40 short-lasting stimuli belonging to one of four sensory modalities (Fig. 2): (i) painful thermo-nociceptive stimuli activating heat-sensitive nociceptors; (ii) non-painful cool stimuli activating cool-sensitive free nerve endings; (iii) non-painful mechanical pinprick stimuli activating mechano-sensitive nociceptors; and (iv) innocuous vibrotactile stimuli activating mechanoreceptors of the medial lemniscus system. Each stimulation block consisted of 40 stimuli.

The interstimulus interval (ISI) was large, variable, and self-paced by the experimenter (5–10 s) to avoid expectation and response habituation. Participants were instructed to keep their gaze fixed on a black cross (3 × 3 cm) placed in front of them on the edge of the bed, at a distance of ~ 2 m, 30° below eye level, for the whole duration of each block. Furthermore, the participants rated the intensity of each stimulus on a numerical rating scale ranging from 0 to 10 (0 was defined as “undetected” and 10 was defined as “maximum intensity imaginable”). At the end of each block, the patients were asked to report whether they had perceived any of the stimuli as painful.

### Sensory stimuli

*Thermo-nociceptive stimuli* consisted of 50-ms pulses of radiant heat generated by a CO_2_ laser (wavelength: 10.6 μm), delivered on the hand dorsum contralateral to the implanted insular electrode. The laser beam was transmitted via an optic fiber, and focusing lenses were used to set the diameter of the beam at the target site to 6 mm. The laser stimulator was equipped with a radiometer providing a continuous measure of the target skin temperature, which was used in a feedback loop to regulate laser power output. The power output of the laser was adjusted to raise the target skin temperature to 62.5 °C in 10 ms and to maintain this temperature for 40 ms. This target temperature was chosen as it was previously shown to be always perceived as clearly painful and pricking^3,4^, and detected with reaction times compatible with the conduction velocity of A-delta fiber nociceptors^38^. To prevent nociceptor fatigue or sensitization, the laser beam was manually displaced after each stimulus^39^. This modality of stimulation was already used in two of our previous studies^3,4^.

*Innocuous cool stimuli* activating cool-sensitive free nerve endings were delivered on the hand dorsum contralateral to the implanted insular electrode using a Thermal Cutaneous Stimulator (TCS) device (QST.Lab, Strasbourg, France), consisting of a control unit connected to a stimulation probe. The stimulation probe has a flat 160 mm^2^ surface in which 16 micro Peltier elements are embedded. The output of the TCS was set to decrease the target skin temperature to 10 °C at a cooling rate of 200 °C/s, for a duration of 250 ms (only in Patient 1, the skin temperature was decreased to 5 °C due to a technical issue). Rapid innocuous skin cooling generated using the TCS was shown to elicit robust cool-evoked potentials at latencies compatible with the conduction velocity of A-delta fibers^40^. To prevent sensitization, the probe was manually displaced after each stimulus.

*Mechano-nociceptive pinprick stimuli* were delivered manually on the volar forearm contralateral to the implanted insular electrode using a custom-built pinprick device, consisting of a holding cylinder containing a freely-sliding steel flat tip probe (diameter: 0.35 mm, uniform geometry) with a calibrated cylindrical weight on top^41^. When applied perpendicular to the skin, the probe and weight slide freely inside the holding cylinder, maintaining a constant normal force (96 mN) entirely determined by the total mass of the probe and the weight. This modality of stimulation was already used in previous studies from the group^41,42^.

*Innocuous vibrotactile stimuli* activating mechanoreceptors of the medial lemniscus system consisted in a 50-ms vibration at 250 Hz delivered via a recoil-type vibrotactile transducer, driven by a standard audio amplifier (Haptuator, Tactile Labs, Canada) and positioned between the fingertips of the index and the thumb contralateral to the implanted insular electrode. The intensity of stimulation was chosen based on results of previous studies showing that the stimuli were systematically perceived as intense, but never painful^3,4,28,43^. This modality of stimulation was already used in two of our previous studies^3,4^.

### Analysis of intracerebral insular recordings in the time domain

For the analysis in the time domain, we used the same procedures as in our previous studies^3–5^. The continuous recordings were filtered using a Butterworth band-pass filter (0.3–40 Hz). 1.5-s epochs were obtained by segmenting the recordings from − 0.5 to 1 s relative to the onset of each stimulus. Epochs contaminated by artefacts were corrected using an independent component analysis (ICA) algorithm^44^ or removed after visual inspection (removed epochs: 2 ± 2 in the thermo-nociceptive modality; 2 ± 3 in the cool modality; 4 ± 1 in the mechano-nociceptive modality; 3 ± 3 in the vibrotactile modality). Separate average waveforms were computed for each subject and stimulus type. Baseline subtraction was performed using the reference interval between − 0.5 and 0 s relative to the onset of each stimulus. Within the averaged waveforms, the peak-to-peak amplitudes of the biphasic waves elicited by each stimulus were used as a measure of the amplitude of the stimulus-evoked LFPs.

We performed a linear mixed model (LMM) analysis of the averaged amplitudes of the phase-locked LFPs using the factors ‘modality’ (4 levels: thermo-nociceptive, cool, mechano-nociceptive, vibrotactile), ‘side’ (2 levels: right insula, left insula), and ‘location’ (2 levels: anterior insula, posterior insula). The contextual variable ‘subject’ was added to the model, to account for the variation of the regression model intercept across participants. Parameters were estimated using restricted maximum likelihood (REML)^45^. Main effects were compared using the Bonferroni confidence interval adjustment.

To isolate the contribution of supramodal and modality-specific neural activities to the low-frequency phase-locked LFPs elicited by the stimuli from the different modalities, we performed, for each patient, a blind source separation using runica^44,46^, an automated form of the Extended Infomax ICA algorithm^47^. When applied to multichannel recordings, the Infomax ICA algorithm separates the signal into a linear combination of independent components (ICs), each having a fixed spatial projection onto the electrode contacts and a maximally independent time course. When ICA is unconstrained, the total number of ICs equals the total number of channels. For each patient, an unconstrained ICA was applied to the average waveforms elicited by each of the modalities of stimulation (thermo-nociceptive, cool, mechano-nociceptive, vibrotactile) obtained on the average of all insular contacts. To estimate the contribution of each obtained IC to the LFPs elicited by the four types of stimuli, the time course of the amplitude of each IC (μV) was expressed as the standard deviation from the mean (z-scores) of the prestimulus intervals (− 0.5 to 0 s) of all four waveforms. If the poststimulus amplitude of an IC was greater than z = 1.5, the IC was considered as reflecting stimulus-evoked activity. We then classified each IC according to its contribution to the four LFP waveforms. For each IC, we computed the ratio between the z-score of a specific modality and the z-scores of the other three modalities^3,28^. If the ratio was ≥ 2.5 for one stimulus modality versus each of the other three modalities, the IC was classified as capturing activity preferential for a specific modality (thermo-nociceptive, cool, mechano-nociceptive, or vibrotactile). If the ratio was ≥ 2.5 for both thermo-nociceptive and cool LFPs versus each of the other two modalities, the IC was classified as capturing activity preferential for thermal modalities. If the ratio was ≥ 2.5 for both thermo-nociceptive and mechano-nociceptive LFPs versus each of the other two modalities, the IC was classified as preferential for nociception. If the ratio was ≥ 2.5 for thermo-nociceptive, cool, and mechano-nociceptive LFPs versus vibrotactile LFPs, the IC was classified as preferential for spinothalamic input. Finally, ICs that contributed to all four LFP waveforms were classified as supramodal. The cutoff value of 2.5 was chosen after testing different cutoff values (1.5, 2, 2.5, and 3) as follows. First, for each sensory modality and participant, two average waveforms using half of the available trials were computed. Second, the ICA was applied on the half datasets, and different classifications were performed using the different cutoff values. The cutoff value of 2.5 provided the most consistent classification across halves, i.e. leading to the same ICs for each class across halves. For each subject, ICs belonging to the same class were then remixed together. The explained variance (EV) of each class of components for the responses of all four modalities was computed as follows: EV = 100 * [1 − (summed squared difference between remixed and raw data)/(summed squared raw data)].

To determine whether the observed low-frequency LFPs were truly originating from the insula, we displayed the signal measured at each insular contact using the average of the two adjacent contacts as a reference (linear current source density)^3^ and identified all the phase reversals occurring within the insula of each patient. The electrical activity generated in a given area can be summarized as an equivalent current dipole, located close to the center of activity, with an orientation orthogonal to the activated cortical surface. Therefore, contacts showing a phase reversal may be considered as located closest to a source of activity, respectively in front and behind the dipole source.

### Analysis of intracerebral insular recordings in the time–frequency domain

For the time–frequency analysis of GBOs, we used the same procedures as in Liberati et al.^4^ The continuous recordings were filtered using a high-pass Butterworth filter (> 20 Hz). 1.5-s epochs were obtained by segmenting the recordings from − 0.5 to 1 s relative to the onset of each stimulus. Epochs contaminated by artifacts were corrected using ICA or removed after visual inspection (removed epochs: 6 ± 3 in the thermo-nociceptive modality; 5 ± 3 in the cool modality; 6 ± 2 in the mechano-nociceptive modality; 6 ± 3 in the vibrotactile modality). A time–frequency representation of each high-pass filtered epoch was obtained using a short-term Fourier transform (STFT) with a fixed 200-ms width Hanning window, chosen to achieve a good tradeoff between time resolution and frequency resolution in the range of gamma-band frequencies^48–50^. The STFT yielded, for each trial, a complex time–frequency spectral estimate *F(t, f)* at each point *(t, f)* of the time–frequency domain plane extending from − 0.5 to 1 s in the time domain, and from 20 to 100 Hz (in steps of 1 Hz) in the frequency domain. After averaging the single-trial time–frequency maps for each subject, the average magnitude of the stimulus-induced changes in oscillation amplitude was estimated as follows^51–53^: *ER%(t, f)* = *[P(t, f) − R(f)]/R(F)* × *100*; where *P(t, f)* =*|F(t, f)|*is an estimate of signal amplitude at each time–frequency point *(t, f)* and *R(f)* is the average amplitude of the signal enclosed within the prestimulus reference interval (− 0.4 to − 0.1 s before the onset of the stimulus), for each estimated frequency, *f*. This yielded, for each insular electrode contact and modality of stimulation, a time–frequency representation of the average stimulus-induced changes of the intracerebral EEG signal (event-related percentage of change in signal power, ER%)^52^.

We performed a LMM analysis of the maximum event-related percentage of change in signal power within the 150–300 ms post-stimulus interval and the 40–90 Hz frequency range—the time interval and frequency range at which stimulus-evoked GBOs were shown to have the greatest amplitude^4^—using the factors ‘modality’ (4 levels: thermo-nociceptive, mechano-nociceptive, cool, vibrotactile), ‘side’ (2 levels: right insula, left insula), and ‘location’ (2 levels: anterior insula, posterior insula). The contextual variable ‘subject’ was added to the model, to account for the variation of the regression model intercept across participants. Parameters were estimated using REML^45^. Main effects were compared using the Bonferroni confidence interval adjustment.

### Analysis of electrocardiographic signals

As in Liberati et al.^4^, we used the EKG to derive heart rate change as a measure of activation of the autonomic nervous system, and therefore as a surrogate measure of stimulus salience^16^. The EKG activity was analyzed offline using Letswave 6. The continuous EKG recordings were filtered using a 0.67–40 Hz band-pass filter, as the 0.67 Hz lower frequency cutoff corresponds to a minimum heart rate of 40 beats per minute (bpm) and the 40 Hz high-frequency cutoff allows eliminating muscle noise^54^. The Pan-Tompkins algorithm was used to recognize the QRS complexes in the EKG signal based on slope, amplitude and width^55^. The time intervals between two QRS complexes were used to generate continuous waveforms expressing the instantaneous heart rate as a function of time. These waveforms were then segmented into 15-s epochs (− 5 to 10 s relative to stimulus onset) and averaged for each participant according to stimulus type (thermo-nociceptive, cool, mechano-nociceptive, and vibrotactile). Finally, within these average waveforms, the difference between the maximum and minimum heart rate value during the 5 s preceding the onset of the stimulus was computed as baseline heart rate change, and the difference between the maximum and minimum heart rate value during the 5 s that followed the onset of the stimulus was computed as an indicator of stimulus-triggered heart rate change. We then performed a LMM analysis to evaluate the effect of the fixed factors ‘modality’ (4 levels: thermo-nociceptive, cool, mechano-nociceptive, and vibrotactile) and ‘time interval’ (2 levels: 5 s before the stimulus and 5 s after the stimulus) on the magnitude of heart rate change. In all analyses, the contextual variable ‘subject’ was added to the LMM models, to account for the variation of the regression model intercept across participants. Parameters were estimated using restricted maximum likelihood (REML)^45^. Main effects were compared using the Bonferroni confidence interval adjustment.

## Supplementary Information

Supplementary Information.

## Data Availability

All supporting data is available at the OSF online repository at the address https://osf.io/tzqru/.

## Abbreviations

EKG: Electrocardiogram
ER%: Percentage of event-related change in oscillation amplitude
EV: Explained variance
fMRI: Functional magnetic resonance imaging
GBO: Gamma-band oscillation
IC: Independent component
ICA: Independent component analysis
iEEG: Intracerebral electroencephalography
LFP: Local field potential
LMM: Linear mixed models
MRI: Magnetic resonance imaging
PET: Positron emission tomography
REML: Restricted maximum likelihood
STFT: Short-term Fourier transform
TCS: Thermal cutaneous stimulator
