# Supplementary material for: Insular responses to transient painful and non-painful thermal and mechanical spinothalamic stimuli recorded using intracerebral EEG
Source: Sci Rep. 2020 Dec 18;10:22319. doi: 10.1038/s41598-020-79371-2 (PMC7749115; doi:10.1038/s41598-020-79371-2)
Supplement: Supplementary file 1 — Supplementary Information. [file 41598_2020_79371_MOESM1_ESM.docx]

**Insular responses to transient painful and non-painful thermal and mechanical spinothalamic stimuli recorded using intracerebral EEG**

Giulia Liberati^1*^, Dounia Mulders^1^, Maxime Algoet^1^, Emanuel N. van den Broeke^1^,

Susana Ferrao Santos^2^, José Géraldo Ribeiro Vaz^3^, Christian Raftopoulos^3^, André Mouraux^1^

^1^Institute of Neuroscience, Université catholique de Louvain

^2^Dept. of Neurology, Saint-Luc University Hospital,

^3^Dept. of Neurosurgery, Saint-Luc University Hospital

| **Subject** | **Hemisphere** | **Contact** | **Description** | **MNI coordinates** |
| --- | --- | --- | --- | --- |
| 1 (electrode 1) | Left | 1 | Posterior insular cortex, external capsule | -33, -12, 13 |
|  |  | 2 | Posterior insular cortex, transition between the long gyrus of the insula and the cortex from the parietal operculum, at the level of the circular gyrus | -38, -12, 14 |
|  |  | 3 | Posterior insular cortex, transition between the long gyrus of the insula and the cortex from the parietal operculum, at the level of the circular gyrus | -44, -12, 17 |
|  |  | 4 | Posterior insular cortex / parietal operculum | -49, -12, 16 |
| 1 (electrode 2) | Left | 1 | Posterior insular cortex, most posterior long gyrus | -38, -9, -5 |
|  |  | 2 | Posterior insular cortex, most posterior long gyrus | -42, -9, -5 |
| 2 | Left | 1 | Anterior insular cortex, adjacent to the opercular portion of the inferior frontal gyrus | -42, 22, -1 |
|  |  | 2 | Anterior insular cortex, adjacent to the opercular portion of the inferior frontal gyrus | -42, 16, -1 |
|  |  | 3 | Anterior insular cortex, second short gyrus | -42, 11, -1 |
|  |  | 4 | Anterior insular cortex, transition between the second and third short gyri | -42, 4, -1 |
|  |  | 5 | Anterior insular cortex, third short gyrus | -42, -1, 0 |
|  |  | 6 | Posterior insular cortex, first long gyrus | -42, -8, 0 |
|  |  | 7 | Posterior insular cortex, transition between the first long gyrus and the second long gyrus | -42, -15, 0 |
|  |  | 8 | Posterior insular cortex, second long gyrus | -42, -19, 0 |
| 3 | Left | 1 | Anterior insular cortex, adjacent to the inferior frontal gyrus | -33, 29, -9 |
|  |  | 2 | Anterior insular cortex, adjacent to the inferior frontal gyrus | -33, 24, -8 |
|  |  | 3 | Anterior insular cortex, adjacent to the inferior frontal gyrus | -34, 19, -7 |
|  |  | 4 | Anterior insular cortex, first short gyrus | -34, 14, -6 |
|  |  | 5 | Anterior insular cortex, second short gyrus | -34, 9, -3 |
|  |  | 6 | Anterior insular cortex, transition between the second and third short gyri | -35, 3, -2 |
|  |  | 7 | Anterior insular cortex, third short gyrus | -35, -2, 0 |
|  |  | 8 | Anterior insular cortex, transition between the third short gyrus and the first long gyrus | -35, -6, 3 |
|  |  | 9 | Posterior insular cortex, first long gyrus | -35, -11, -5 |
|  |  | 10 | Posterior insular cortex, first long gyrus | -35, -16, 6 |
|  |  | 11 | Posterior insular cortex, transition between the first long gyrus and the second long gyrus | -35, -21, 8 |
|  |  | 12 | Posterior insular cortex, transition between the first long gyrus and the second long gyrus | -35, -26, 11 |
| 4 | Left | 1 | Anterior insular cortex, first short gyrus | -35, 14, -1 |
|  |  | 2 | Anterior insular cortex, adjacent to the opercular portion of the inferior frontal gyrus | -39, 14, -3 |
|  |  | 3 | Anterior insular cortex, adjacent to the opercular portion of the inferior frontal gyrus | -44, 13, -6 |
| 5 | Right | 1 | Anterior insular cortex, adjacent to the inferior frontal gyrus | 40, 23, -6 |
|  |  | 2 | Anterior insular cortex, second short gyrus | 40, 13, -2 |
|  |  | 3 | Anterior insular cortex, second short gyrus | 40, 9, 0 |
|  |  | 4 | Anterior insular cortex, third short gyrus | 40, 4, 1 |
|  |  | 5 | Anterior insular cortex, third short gyrus | 40, 0, 3 |
|  |  | 6 | Anterior insular cortex, third short gyrus | 40, -5, 5 |
|  |  | 7 | Posterior insular cortex, first long gyrus | 40, -10, 7 |
|  |  | 8 | Posterior insular cortex, first long gyrus | 40, -15, 9 |
|  |  | 9 | Posterior insular cortex, first long gyrus, adjacent to the transverse temporal gyrus | 40, -20, 11 |
|  |  | 10 | Posterior insular cortex, transition between the first long gyrus and the second long gyrus, adjacent to the transverse temporal gyrus | 40, -24, 12 |
|  |  | 11 | Posterior insular cortex, second long gyrus, adjacent to the transverse temporal gyrus | 40, -30, 15 |
| 6 (electrode 1) | Right | 1 | Anterior insular cortex, first short gyrus | 26, 22, -12 |
|  |  | 2 | Anterior insular cortex, first short gyrus | 26, 19, -8 |
|  |  | 3 | Anterior insular cortex, second short gyrus | 26, 14, -6 |
|  |  | 4 | Anterior insular cortex, second short gyrus | 26, 2, 5 |
|  |  | 5 | Anterior insular cortex, third short gyrus | 26, 1, 9 |
|  |  | 6 | Anterior insular cortex, third short gyrus | 26, -4, 12 |
|  |  | 7 | Posterior insular cortex, first long gyrus | 26, -8, 16 |
|  |  | 8 | Posterior insular cortex, first long gyrus | 26, -13, 18 |
|  |  | 9 | Posterior insular cortex, first long gyrus | 26, -17, 22 |
|  |  | 10 | Posterior insular cortex, first long gyrus | 26, -20, 27 |
| 6 (electrode 2) | Right | 1 | Anterior insular cortex, ventral portion of the first short gyrus | 36, 17, -17 |
|  |  | 2 | Anterior insular cortex, ventral portion of the first short gyrus | 38, 15, -15 |
| 7 | Right | 1 | Anterior insular cortex, ventral portion of the intersection between the first long gyrus and the second long gyrus | 44, 15, -9 |

**Suppl. 1. Localization and MNI coordinates of insular electrode contacts**

**
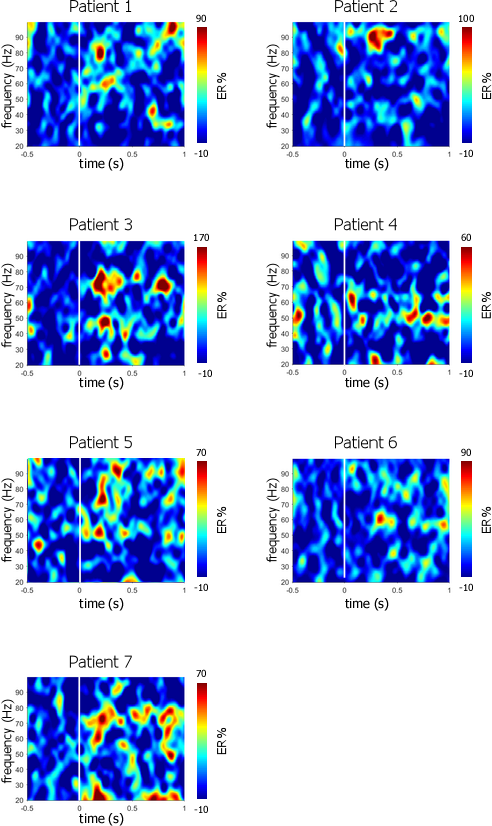
**

**Suppl. 2. Time-frequency representation of the changes in oscillatory power (20-100 Hz) recorded at the insular contacts in which, for each patient, GBOs elicited by thermo-nociceptive stimuli were more pronounced (ER%).** See Fig. 1 for the group level percentage of change.
